# C-Type Natriuretic Peptide: A Multifaceted Paracrine Regulator in the Heart and Vasculature

**DOI:** 10.3390/ijms20092281

**Published:** 2019-05-08

**Authors:** Amie J. Moyes, Adrian J. Hobbs

**Affiliations:** William Harvey Research Institute, Barts and The London School of Medicine & Dentistry, Queen Mary University of London, Charterhouse Square, London EC1M 6BQ, UK; a.j.hobbs@qmul.ac.uk

**Keywords:** natriuretic peptide, vascular, endothelial cell, cardiomyocyte, fibroblast, inflammation, heart failure, hypertension, angiogenesis

## Abstract

C-type natriuretic peptide (CNP) is an autocrine and paracrine mediator released by endothelial cells, cardiomyocytes and fibroblasts that regulates vital physiological functions in the cardiovascular system. These roles are conveyed via two cognate receptors, natriuretic peptide receptor B (NPR-B) and natriuretic peptide receptor C (NPR-C), which activate different signalling pathways that mediate complementary yet distinct cellular responses. Traditionally, CNP has been deemed the endothelial component of the natriuretic peptide system, while its sibling peptides, atrial natriuretic peptide (ANP) and brain natriuretic peptide (BNP), are considered the endocrine guardians of cardiac function and blood volume. However, accumulating evidence indicates that CNP not only modulates vascular tone and blood pressure, but also governs a wide range of cardiovascular effects including the control of inflammation, angiogenesis, smooth muscle and endothelial cell proliferation, atherosclerosis, cardiomyocyte contractility, hypertrophy, fibrosis, and cardiac electrophysiology. This review will focus on the novel physiological functions ascribed to CNP, the receptors/signalling mechanisms involved in mediating its cardioprotective effects, and the development of therapeutics targeting CNP signalling pathways in different disease pathologies.

## 1. Introduction

The natriuretic peptides are a family of three structurally related hormones that play unique and distinctive roles within the cardiovascular system. The physiological functions of atrial natriuretic peptide (ANP) and brain natriuretic peptide (BNP) have been intensively investigated over the past few decades, however there has been considerably less focus on C-type natriuretic peptide (CNP). ANP and BNP are expressed in the heart [1,2,3,4] and are released in response to a volume-induced stretch of the atria and ventricles, respectively [5,6]. These peptides act as endocrine hormones and contribute to the regulation of cardiac structure, blood pressure and blood volume [7]. In contrast, the tissue distribution and mode of action of CNP is different, with recent studies revealing diverse endogenous roles of CNP including the control of vascular tone, leukocyte activation, angiogenesis, smooth muscle and endothelial cell proliferation, vascular integrity, coronary blood flow, cardiac fibrosis, cardiac hypertrophy, and electrophysiology. These aspects of CNP physiology and pathology will be detailed herein.

## 2. CNP Expression, Release & Degradation

CNP is a 22 amino acid peptide that is produced following the processing of preproCNP by a signal peptidase and subsequent cleavage of proCNP by the endoprotease furin [8]. Two forms of CNP exist in tissue and plasma, CNP-53 and CNP-22 [8], although the protease responsible for processing the elongated peptide into its shorter, more prevalent form is not known. CNP-22 was initially discovered in extracts from porcine brain [9]. In addition to its abundant expression in the CNS, high levels of CNP are found in chondrocytes [10] and endothelial cells [11,12], which constitutively release the peptide. Other cells within the cardiovascular system, including cardiomyocytes [13,14] and fibroblasts [15], also produce CNP, however, tissue expression and plasma levels are relatively low in healthy individuals, suggesting that CNP most likely acts as a local paracrine/autocrine mediator in the heart and blood vessels. 

The half-life of CNP in plasma is short (2.6 min) [16], therefore, degradation is rapid which may account for the low concentrations (fmol–pmol range) of the peptide measured in the circulation [11,17]. There are two main pathways by which CNP is inactivated, cleavage by neutral endopeptidase (NEP) [18], or internalisation by natriuretic peptide receptor C (NPR-C) followed by endocytosis and lysosomal degradation [19,20]. Overall, the contribution of each pathway to the degradation of natriuretic peptides appears to be equal [21] in healthy subjects but there is evidence to suggest that during pathophysiological conditions where natriuretic peptide levels are raised and NPR-C may be saturated, NEP may play a major role in clearance [22]. Furthermore, the tissue distribution of NEP and/or NPR-C may affect CNP inactivation in different organs, for example, CNP is internalised more readily by NPR-C in the kidney compared to the lungs [23].

Most of the stimuli that are known to increase gene expression and/or trigger the release of CNP are pertinent to cardiovascular health including shear stress [24,25], inflammatory cytokines such as tumour necrosis factor (TNF)-α [26], interleukin (IL)1β [26,27], transforming growth factor (TGF)-β [12,28], and bacterial lipopolysaccharide [26,29]. In accordance with these findings are studies showing that plasma levels of CNP are elevated in patients with heart failure (HF) [30] and sepsis [31]. In contrast, CNP release is attenuated by oxidised low-density lipoprotein [32] and vascular endothelial growth factor [33]. 

## 3. Natriuretic Peptide Receptors

CNP exerts its biological effects via the activation of two cell surface receptors, natriuretic peptide receptor B (NPR-B, also termed guanylyl cyclase-B, GC-B) and natriuretic peptide receptor C (NPR-C) [34,35]. The peptide has a very low binding affinity for natriuretic peptide receptor A (NPR-A) [36], which is the endogenous receptor for the ligands ANP and BNP. Both NPR-B and NPR-C are widely expressed and are found on endothelial cells [37,38], smooth muscle cells [37,39], cardiomyocytes [14,40], and fibroblasts [15,41]. NPR-C is the most abundant natriuretic peptide receptor and accounts for ~95% of the total natriuretic peptide receptor population in endothelial cells [42]. 

CNP has a similar binding affinity for NPR-B and NPR-C [36] but the signalling pathways activated by each receptor are markedly different. NPR-B is a particulate guanylyl cyclase receptor that catalyses the conversion of guanosine-5′-triphosphate to cyclic guanosine-3′,5′-monophosphate (cGMP), a second messenger that activates protein kinase G I and II [43,44,45], which in turn alters cellular functions by phosphorylating specific target proteins. NPR-C was originally considered to be a clearance receptor [46] devoid of signalling activity but later it was shown to contain pertussis toxin sensitive Gi binding domains within the intracellular C-terminal tail that couple to adenylyl cyclase inhibition (by G_i_ α subunit) and phospholipase C-β activation (by G_i_ βγ subunits) [47,48,49,50]. Two subtypes of NPR-C have been reported with different molecular masses, a 67-kDa protein and a 77-kDa protein, but it is not known if their capacity to signal and clear natriuretic peptides in vivo is distinct [51,52]. A study in isolated rat glomerular membranes showed that the 67-kDa NPR-C has a high affinity for CNP and activation of this subtype reduces cAMP synthesis via G_i_ signalling, whereas the 77-kDa receptor has a very low affinity for CNP and is involved in ligand internalization [48,53].

## 4. CNP Regulates Vascular Tone and Blood Pressure

Pharmacological experiments on isolated blood vessel preparations have shown that CNP is a potent vasodilator of conduit and resistance arteries throughout the vascular tree [54,55,56,57,58,59,60,61,62,63,64,65,66]. In the microvasculature, CNP is more efficacious than ANP and BNP, suggesting it may play a role in regulating peripheral vascular resistance [64,67]. Numerous studies have shown that CNP infusion reduces systemic blood pressure in both humans and animals [68,69,70,71,72]. Despite this, the physiological role of endogenous CNP in the cardiovascular system had not been elucidated until recently. Early studies of global CNP knockout (KO) mice were confounded by the effects of CNP deletion on bone development [10]. These animals exhibit skeletal abnormalities, dwarfism, and a high mortality rate, thus, investigations utilising the Cre/Lox recombination system to generate animals with cell-restricted deletion of CNP have been key to gaining a fuller understanding of the function of this peptide in vivo. Endothelial-specific deletion of CNP in mice results in elevated blood pressure and impaired responses to endothelium-dependent vasodilators, providing definitive evidence that the constitutive release of CNP contributes to the regulation of vascular tone [73,74,75]. The (patho)physiological relevance of these experimental findings is exemplified by the discovery of polymorphisms in the CNP and furin genes that are associated with hypertension in humans [76,77].

CNP-mediated vasodilation occurs via different mechanisms depending on the species, vessel studied, and/or natriuretic peptide receptor activated. In conduit arteries, CNP-induced relaxation is blocked by the dual NPR-A/B antagonist HS-142-1, suggesting NPR-B activation and subsequent production of cGMP mediates the dilatory effects of CNP in large vessels [57,60,63]. However, in the resistance vasculature the importance of NPR-C in the vasoreactivity of CNP increases. In both rodents and humans, a similar pathway exists involving activation of NPR-C and smooth muscle cell hyperpolarisation [66,71,78,79,80]. In the rat mesenteric artery, the release of CNP accounts for a major component of the endothelium-derived hyperpolarisation (EDH) induced by acetylcholine, a response that can be inhibited by NPR-C antagonists, blockade of small and intermediate conductance calcium-sensitive potassium channels (SK_Ca_ and IK_Ca_), and G-protein inwardly rectifying potassium channels (GIRK) [65]. It is proposed that the opening of SK_Ca_ and IK_Ca_ on the endothelial cell triggers the release of CNP that binds to NPR-C on the smooth muscle cell resulting in the G_i/0-_mediated activation of GIRK, potassium efflux, and hyperpolarisation. In human arteries, both GIRK and large conductance calcium-activated K^+^ channels (BK_Ca_ channels) have been implicated in CNP-evoked vasodilation [79]. Alternatively, studies in rats have shown that NPR-C can also couple to eNOS, resulting in the production of nitric oxide, although, this mechanism has only been reported in larger diameter vessels [81,82].

The receptor that mediates the endogenous regulatory effects of endothelial-derived CNP on vascular tone in vivo is still under debate. Global and smooth muscle-specific NPR-B KO mice are normotensive and their vascular function is normal despite the vasodilator responses to exogenous CNP being impaired [74,83]. It has been proposed that CNP maintains endothelial function independently of smooth muscle NPR-B and that alterations in the production of the vasoconstrictor endothelin-1 account for the elevations in blood pressure observed in ecCNP KO animals, however, the mechanism(s) involved has not been elucidated. A recent study proposed that NPR-B signalling in pericytes may be more important than vascular smooth muscle. This latest research shows that the disruption of NPR-B under the control of the PDGFRβ promotor in mice results in a hypertensive phenotype, indicating CNP may participate in paracrine communication between endothelial cells and pericytes to regulate peripheral vascular resistance [75]. 

In contrast, accumulating evidence suggests that NPR-C mediates a large proportion of the vasodilator effects of CNP in the vasculature. NPR-C KO mice exhibit impaired endothelial function and a diminished hypotensive response to CNP in vitro and in vivo [73]. The original publication describing global deletion of NPR-C in mice reports a lower blood pressure in these animals (males only). However, more recently it was shown that female NPR-C KO exhibit elevated blood pressure and diminished vascular endothelial function [73,84]. This discrepancy may be due to sex differences in the clearance versus signalling functions of NPR-C in mice, however, data from human genome wide association studies linking variants of the NPR-C gene with hypertension did not find a disparity between sexes, suggesting the NPR-C signalling pathway is equally important in men and women [85]. Patients with the blood pressure elevating NPR-C genotype have lower levels of receptor mRNA and protein in their vascular smooth muscle cells, supporting the theory that diminished CNP activation of NPR-C may underlie this association (as opposed to altered clearance) [86]. Further evidence in favour of a role of NPR-C in the pathogenesis of hypertension was published in a study investigating the effects of the endogenous secretory peptide musclin (also known as osteocrin). Musclin is a competitive ligand at NPR-C and the infusion of musclin increases systolic blood pressure in vivo [87]. Gene expression levels of musclin are elevated in spontaneously hypertensive rats (SHR) and the administration of anti-musclin antibodies reduces blood pressure in these animals, intimating that interference with NP binding at NPR-C by musclin may contribute to the hypertensive state of these animals [87]. However, contrary to this research is a study showing that the infusion of musclin (osteocrin) lowers blood pressure in mice, an effect that is absent in NPR-A KO animals, suggesting that an increase in circulating levels of ANP and/or BNP due to the blockade of peptide clearance by NPR-C accounts for this response [88]. It is possible that musclin interferes with both the signalling and clearance functions of NPR-C and plays a different role in the regulation of blood pressure in healthy and diseased animals. Indeed, the vasoconstrictor and blood pressure elevating effects of musclin are significantly enhanced in SHR compared to normotensive controls, intimating that a change in receptor expression or the signalling/clearance function of NPR-C occurs in hypertension that alters the response to musclin in this model of disease [87]. This is further supported by data demonstrating that NPR-C agonism in SHR attenuates the development of high blood pressure, an effect that is not observed in control Wistar-Kyoto rats [89].

## 5. CNP Influences Vascular Remodelling and Promotes Angiogenesis

CNP has direct effects on the mitogenesis of endothelial and smooth muscle cells and it promotes wound healing and vascular repair by stimulating endothelial growth, whilst concomitantly inhibiting smooth muscle cell proliferation. This dual protective role of CNP was first described in animal models of vein graft and balloon angioplasty, clearly showing that CNP treatment accelerates re-endothelialisation and reduces deleterious neointimal hyperplasia [90,91,92]. A similar response to CNP has been observed in carotid arteries subjected to physical damage [93]. Many of these studies report an increase in cGMP production following treatment with CNP [91,93,94,95], intimating the involvement of NPR-B, however, others have shown that CNP influences the growth of endothelial and smooth muscle cells via NPR-C in a cGMP-independent manner. These experiments revealed that the pro- and anti-mitogenic effects of CNP are mediated by the extracellular signal-related kinase (ERK) 1/2 and can be blocked by the NPR-C antagonist, M372049, and by the G_i/0_ inhibitor, *Pertussis* toxin, despite significant increases in cGMP production by both cell types [37,96]. Activation of ERK 1/2 by CNP results in the enhanced expression of cell cycle promotors (cyclin D1) in endothelial cells and inhibitory cell cycle proteins in smooth muscle cells (p21 and p27). This is further supported by the observation that primary microvascular lung endothelial cells, isolated from NPR-C KO mice, proliferate more slowly than wildtype (WT) cells, whilst aortic smooth muscle cells, isolated from KO animals, grow at a faster rate [37]. Indeed, in vivo studies show that mice lacking endothelial-derived CNP and NPR-C exhibit slower wound healing and greater intimal hyperplasia following vascular injury, indicating that vascular CNP release is a vital step in tissue repair [97].

The ability of CNP to influence endothelial cell growth led researchers to question the role of this peptide in angiogenesis. The potential angiogenic effects of CNP were initially tested in classical assays of endothelial tube formation in vitro and revealed that CNP-induced increases in capillary network formation are of a similar magnitude to the potent pro-angiogenic mediator, VEGF [98]. In addition to this, the gene transfer of CNP directly into ischaemic muscle has been reported to enhance blood flow recovery and increase capillary density following ligation and excision of the femoral artery in mice [98]. Research concurs that these angiogenic responses are dependent on the activation of ERK 1/2, however, there are opposing data published regarding the receptor involved.

A comprehensive study performed in KO animals suggests that the endogenous effects of endothelial-derived CNP on angiogenesis are mediated by NPR-C, whereas both receptors are implicated when CNP is administered pharmacologically. For example, branching angiogenesis in human umbilical vein endothelial cells (HUVEC) has been shown to be blocked by an inhibitor of cGMP-dependent protein kinase, suggesting the involvement of NPR-B signalling [98]. In contrast, tube formation in murine pulmonary endothelial cells is inhibited by *Pertussis* toxin and NPR-C antagonism [97]. Experiments performed in transgenic mice show that basal endothelial tubule formation, de novo aortic sprouting, and restoration of blood flow following hindlimb ischaemia is diminished in ecCNP KO and NPR-C KO tissues/animals, whilst NPR-B KO display a similar angiogenic capacity to WT mice [97]. In addition to this, the same study reported that patients with critical limb ischaemia have lower levels of CNP and NPR-C in biopsies of the gastrocnemius muscle, suggesting that diminished signalling via this pathway may contribute to the insufficient angiogenic response to hypoxia associated with peripheral arterial disease. 

While the majority of studies indicate that CNP promotes angiogenesis, there is also evidence demonstrating that the NPR-C agonist cANF^4-23^ reduces neovascularization in murine sponge implants [99]. This finding was accompanied by reduced levels of VEGF which corroborates with other studies showing that CNP and cANF^4-23^ inhibit VEGF expression and signalling in vascular smooth muscle and endothelial cells [100]. Contrary to this, VEGF has also been shown to reduce CNP secretion from cultured endothelial cells [33], suggesting there may be a reciprocal relationship between the two vascular mediators, however, it is not known if an interplay between the two factors modulates angiogenesis. 

## 6. CNP Inhibits Inflammation and Slows the Development of Atherosclerosis

The first indication that CNP may influence the inflammatory response to infection and disease comes from research showing that the cytokines IL-1α, IL-1β, and tumour necrosis factor (TNF)α stimulate the release of CNP from endothelial cells [26,27]. The most potent of these cytokines (at inducing CNP secretion) is TNFα, which is released by macrophages during the acute phase of inflammation. Another strong stimulus for triggering CNP release from the endothelium is the endotoxin bacterial lipopolysaccharide (LPS) [26,29]. Indeed, CNP levels are markedly increased in patients with septic shock, a 5–10-fold increase in plasma CNP concentrations has been reported in several studies [31,101,102,103]. Furthermore, plasma concentrations of NT-proCNP are strongly associated with inflammation-induced organ dysfunction and are predictive of a detrimental outcome [101,104]. It has also been suggested that measurement of NT-proCNP in the early phase of septic shock might help to predict the emergence of sepsis-induced encephalopathy [103]. Together, these data suggest that the acute release of CNP may modulate the progression of sepsis and other inflammatory disorders.

Endothelial activation by inflammatory mediators is a key event in the pathogenesis of sepsis and cardiovascular diseases such as atherosclerosis. Changes in the expression of cell adhesion molecules, such as integrins and selectins, facilitate the recruitment and adherence of leukocytes during the initial phase of the immune response [105,106]. CNP has been shown to dampen endothelial activation induced by range of inflammatory stimuli both in vitro and in vivo. IL-1β and histamine-induced leukocyte rolling in murine post-capillary venules is inhibited by CNP and cANF^4-23^ via the suppression of P-selectin expression [107]. CNP infusion via mini-pump inhibits LPS-stimulated leukocyte infiltration into the lungs, attenuates E-selectin gene expression, and reduces the levels of the inflammatory mediators TNFα, macrophage inflammatory protein-2, monocyte chemoattractant protein-1 (MCP-1), and interleukin-6 (IL-6) [108]. In addition, CNP inhibits elevations of intercellular adhesion molecule-1, vascular cell adhesion molecule-1, E-selectin, and P-selectin expression in HUVECs stimulated with LPS [109]. The mechanism by which CNP attenuates this response at least in vitro is via the inhibition of pro-inflammatory NF-κB and p38 signalling pathways and the activation of the pro-survival PI3K/Akt pathway. 

Endothelial-derived CNP also appears to maintain a resting anti-inflammatory influence on the vascular wall as ecCNP KO mice exhibit greater leukocyte rolling at baseline prior to stimulation with an inflammogen [73]. In addition, the response to LPS and TNFα-induced peritonitis is significantly exacerbated in these animals. The anti-leukocyte effects of the endogenous peptide may involve similar mechanisms to those induced by an exogenous application of the peptide, as higher vascular P-selectin expression is observed in mice lacking endothelial CNP. Given that a similar increase in leukocyte recruitment was observed in NPR-C KO mice following treatment with LPS, it is hypothesised that NPR-C-driven suppression of cell adhesion molecule expression may underpin the immune dampening effect of CNP [73]. This facet of CNP biology is clearly important in the context of atherosclerosis as genetic ablation of CNP leads to an increase in the development of atherosclerotic lesions, greater infiltration of macrophages, and the formation of aortic and abdominal aneurysms in ecCNP/ApoE double-KO mice [73]. Indeed, this finding fits with previous work demonstrating reduced CNP immunoreactivity in diseased human coronary arteries and the inverse relationship discovered between the expression of CNP and lesion severity [110]. Moreover, CNP inhibits the proliferative and pro-migratory effects of oxidised LDL on smooth muscle cells which may affect the growth and stability of atherosclerotic plaques [111]. The spontaneous development of aneurysms in ApoE KO mice is rare, however, ecCNP/ApoE double-KO mice are more susceptible to this phenomenon, suggesting that CNP may help to maintain the structural integrity of the vessel wall [73]. Interestingly, aneurysms were only observed in male double-KO mice, this observation aligns with the human condition that predominantly affects the elderly male population [112]. It is possible that CNP regulates the expression and release of matrix metalloproteinases (MMPs) which are implicated in the development of aneurysms. In support of this thesis are data showing that CNP modulates the expression of MMP-2 and MMP-9 in chondrocytes and the kidney [113,114]. 

There are a number of other inflammatory disorders where CNP has proven beneficial in experimental models of disease. CNP reduces the number of macrophages, neutrophils, and lymphocytes accumulating in the lungs of mice exposed to bleomycin in a model of pulmonary hypertension [115]. In a rat model of haemorrhagic shock, CNP reduces markers of oxidative stress and the expression of tumour necrosis factor (TNF)-α, interleukin (IL)-6, and IL-1β in the kidney, suggesting it may improve symptoms of acute renal injury associated with this condition [116]. Furthermore, studies performed using transgenic mice overexpressing CNP in endothelial cells suggest that CNP regulates inflammation associated with obesity. Overexpression of endothelial-derived CNP improves glucose tolerance, decreases insulin resistance, and inhibits adipose macrophage infiltration in mice that are fed a high-fat diet [117]. Using the same animals, these authors also demonstrate that CNP inhibits expression of inflammatory markers IL-6, MCP-1, and CD68 in the liver of mice fed high fat diets in a model of non-alcoholic steatohepatitis [118]. Thus, the anti-inflammatory benefits of endothelial-derived CNP are not entirely limited to cardiovascular disease. 

## 7. CNP is a Novel Regulator of Cardiac Structure and Function

### 7.1. CNP and HF

For many years the role of CNP in the heart was largely ignored as the majority of research focused on the cardiac hormones ANP and BNP. In addition to this, the expression of CNP in cardiomyocytes is much lower than that of ANP and BNP, suggesting that under basal conditions it does not play a major role in regulating cardiac function [14,119]. However, it has been widely reported that cardiac gene expression and plasma levels of CNP are increased in patients with HF [13,30,120,121,122,123]. Elevated circulating levels of CNP are associated with a high-risk phenotype in patients with cardiovascular comorbidities and left ventricular dysfunction [17]. Furthermore, plasma NT-pro CNP levels in patients with HF are correlated with disease severity and are a strong predictor of all cause mortality and hospitalization in patients with HF with preserved ejection fraction (HFpEF) [124]. Yet these studies do not tell us if CNP is produced by the heart or by other organs during HF. A key experiment comparing plasma CNP levels in the coronary sinus and aortic root of failing hearts discovered that concentrations of CNP are significantly higher in the coronary bloodstream than those measured in the systemic circulation, providing the first direct evidence that CNP is released by the heart in HF [125].

In non-failing hearts, levels of CNP are higher in the atria than in the ventricles but studies in mini-pigs have shown that cardiac pacing induces a 15-fold increase in CNP expression in the ventricles along with elevated levels of CNP protein, demonstrating that acute cardiac stress elicits immediate upregulation of the gene and an increase in CNP release [13]. There also appears to be a switch between natriuretic peptide signalling in the failing ventricle. In sham hearts, ANP induces a greater increase in guanylyl cyclase activity than CNP, however, in pressure overload-induced HF, CNP elicits twice as much cGMP production than ANP. This might be due to a reduction in NPR-A expression, suggesting that CNP signalling via NPRB may be more important during HF [126]. However, other studies have reported that NPR-B expression decreases in the ventricles of the failing heart [13,127], whereas NPR-C increases are the most pronounced of the three NP receptors in end-stage disease [128].

### 7.2. CNP Directly Modulates Cardiomyocyte Contractility, Fibrosis, And Hypertrophy

CNP has been shown to exert direct effects on cardiac contractility, although both positive and negative inotropic responses have been reported. For example, CNP increases myocyte contractile force in canine isolated atrial and ventricular preparations [129,130], whereas positive lusitropic and negative inotropic effects have been observed in rat heart muscle preparations [131,132]. A number of studies have shown that CNP induces phosphorylation of the sarcoplasmic reticulum calcium pump (SERCA) 2 regulator, phospholamban (PLB), and cardiac troponin I (cTnI), a regulatory protein that controls the calcium-mediated interaction between actin and myosin [131,132,133,134]. The positive lusitropic and inotropic effects of CNP reported in the failing rat heart are associated with phosphorylation of both of these regulatory proteins in addition to an increase in sarcoplasmic reticulum (SR) calcium load [132]. Further investigations demonstrated that this negative inotropic effect of CNP is sensitive to PKG inhibition (i.e., NPR-B-dependent), SERCA2 inhibition, and is absent in SERCA2 KO mice [135]. It is proposed that an increase in SERCA2 activation via phosphorylation of PLB by CNP causes a higher fraction of the cytosolic Ca^2+^ to be sequestered back into the SR, therefore reducing Ca^2+^ activation of the myofilaments resulting in a negative inotropic effect. In contrast, others have demonstrated that CNP induces a positive inotropic response in the heart and that PLB phosphorylation results in a greater uptake of Ca^2+^ into the SR, creating a larger pool of Ca^2+^ available for contraction [134]. 

Biphasic responses to CNP have also been reported by a number of studies, where an initial, transient positive inotropic response is followed by a slow developing reduction in contractility [133,135,136]. The nature of this biphasic response was investigated to elucidate if the two opposing effects were due to activation of different receptors, however it appears that both phases of the response are mediated by NPR-B as a cGMP analogue mimicked both the immediate and delayed phase of the contractile response. The NPR-C agonist cANF^4-23^ did not affect contractility and no changes in cardiac cAMP were observed [133]. The reason why there is such ambiguity in the contractile responses elicited by CNP could be attributed to cross-talk between the cGMP and cAMP signalling systems in the heart. It has been shown that the negative inotropic response to CNP is the dominating effect when the cAMP signalling is reduced (e.g., during β-adrenoceptor blockade), whilst the effect is completely lost in the presence of maximal β-adrenoceptor stimulation by isoprenaline [135]. In both failing and non-failing hearts, CNP increases the positive inotropic effect of β-adrenoceptor stimulation due to cGMP inhibition of phosphodiesterase (PDE)3, an enzyme responsible for the breakdown of cAMP produced during β-adrenoceptor stimulation [137,138]. Thus, the contractile effect of CNP observed in different experimental models is likely to be influenced by intracellular cAMP levels and β-adrenoceptor stimulation. NPR-C signalling may also influence cardiac contractility, although this has not been directly investigated, receptor stimulation would likely result in a reduction in cAMP via the inhibition of adenylyl cyclase [50,139]. cANF^4-23^ has also been shown to inhibit L-type calcium currents in atrial myocytes [140], if a similar pathway is present in ventricular myocytes NPR-C activation may induce a negative inotropic response. 

Cardiac remodelling during HF is characterised by fibroblast proliferation, myofibroblast transformation, and collagen deposition resulting in the development of cardiac fibrosis. This leads to ventricular distortion and myocardial stiffness, which has significant consequences for heart function [141]. CNP exerts anti-fibrotic effects in the heart and is significantly more potent at reducing fibroblast growth and extracellular matrix production than other natriuretic peptides [15]. CNP is expressed and released by cardiac fibroblasts in response to the basic fibroblast growth factor (BFGF), TGFβ, and endothelin 1. It induces a greater increase in cGMP and more suppression of collagen synthesis than ANP and BNP [15]. In addition, fibroblast differentiation, migration, and the production of the pathologic mediators, MCP-1 and PAI-1, are attenuated by CNP in vitro [128,142,143]. 

In addition to attenuating fibrosis, CNP also exerts anti-hypertrophic effects in the heart. Experiments performed in isolated rat cardiomyocytes have shown that CNP inhibits basal and endothelin-1-induced protein expression, ANP secretion, and the expression of the hypertrophic genes GATA-4 and MEF-2. Endothelin-1-induced increases in calcium/calmodulin-dependent kinase and ERK activities are also attenuated by CNP. These effects are recapitulated using a cGMP analogue, suggesting that the mechanism involves activation of NPR-B [144]. Similarly, CNP has been shown to reduce angiotensin II-induced increases in murine cardiomyocyte size, indicating that CNP directly supresses hypertrophic signalling cascades [145].

## 8. Endogenous CNP Is Cardioprotective in Animal Models of Heart Failure

The generation of tissue-specific knockouts has facilitated a greater understanding of the cell types responsible for CNP release in the heart and how each source of CNP impacts cardiac structure and function in disease. At baseline, mice lacking cardiomyocyte- (cmCNP KO) and fibroblast-derived CNP (fbCNP KO) exhibit no overt changes in cardiac contractility, structure, or fibrosis, confirming previous speculation that CNP plays a minimal role in healthy hearts [145]. However, following aortic banding (pressure overload-induced HF) both cmCNP KO and fbCNP KO mice display a greater decline in ejection fraction, increased ventricular dilation, greater cardiac hypertrophy (cmCNP KO only), and more collagen deposition compared to littermate controls. In contrast, endothelial-derived CNP does not appear to contribute to cardioprotection, at least in this model. Thus, endogenous CNP secreted from cardiomyocytes and fibroblasts reduces the deleterious pathological changes that occur during heart failure. Comparable cardiac dysfunction, hypertrophy, and fibrosis is observed in NPR-C KO animals subjected to aortic banding, suggesting that NPR-C mediates the effects of CNP in myocytes and fibroblasts. Indeed, CNP infusion via mini-pump reverses cardiac dysfunction and fibrosis during HF in WT animals but not NPR-C KO mice. cmCNP KO animals fared worse than WT animals upon stimulation with isoprenaline (i.e., sympathetic hyperactivation models of HF), whilst the loss of NPR-B did not adversely affect the hypertrophic or fibrotic response [145]. This contrasts with previous studies that show transgenic rats expressing a dominant negative form of NPR-B exhibit cardiac hypertrophy at baseline [146]. However, these mutants do not exhibit cardiac fibrosis, nor changes in contractile function before or after chronic volume overload, so perhaps, in the longer-term, NPR-B plays a predominant role in regulating compensatory hypertrophy, whereas NPR-C regulates the maladaptive hypertrophy and anti-fibrotic effects of CNP. In support of this are data showing that cardiomyocyte-specific NPR-B deletion does not alter the response to pressure overload-induced HF in mice [147], intimating that endogenous NPR-B signalling is either not vital in pathologic remodelling in the heart, or another system compensates for the loss of NPR-B. However, NPR-B heterozygote mice are susceptible to aortic stenosis [148], suggesting that the importance of this NPR subtype might sit outside the cardiomyocyte. This does not mean that NPR-B cannot be targeted pharmacologically, as recent studies suggest that novel designer peptides that bind to NPR-B can reduce fibroblast proliferation and collagen secretion in vitro and in vivo [128,149,150]. This role of endogenous NPR-C signalling in regulating fibrosis is supported by other studies that have observed greater cardiac dysfunction, atrial collagen deposition, and higher levels of TGFβ and TIMP1 in NPR-C KO mice subjected to angiotensin II-induced pressure overload [151,152]. Furthermore, a functional genetic variant in NPR-C has been discovered in humans that is associated with diastolic dysfunction. This single nucleotide polymorphism (SNP) does not affect the protein expression of NPR-C or the circulating plasma levels of natriuretic peptides, suggesting that downstream signalling is affected. It is postulated that this SNP leads to dysfunction of the catalytic domain of NPR-C, and that aberrant signalling in fibroblasts contributes to cardiac fibrosis and impaired diastolic function [153]. The precise mechanism by which NPR-C signalling inhibits fibroblast proliferation/collagen synthesis is unknown, but it has been shown that CNP- and cANF^4-23^-mediated stimulation of NPR-C can activate a non-selective cation current that is partly carried by transient receptor potential C channels, and the authors tentatively suggest that this may affect the secretory state of the cell [154].

However, it appears that NPR-C may play a Janus-faced role in HF as other work suggests that the removal/blockade of the clearance function of NPR-C is beneficial in cardiac disease. NPR-C KO mice cross-bred with animals that spontaneously develop atrial fibrosis (TGFβ1 overexpression) display significantly less fibrosis and collagen deposition than controls. Also, NPR-C knockdown in cultured fibroblasts stimulated with TGFβ1 results in a lower expression of pro-fibrotic markers pSmad and collagen. These effects are reversed by NPR-A knockdown, intimating that the reduced clearance of ANP and the subsequent increase in ANP signalling underlies this effect [88,155]. In addition, transgenic mice overexpressing osteocrin (OSTN-Tg) have an improved prognosis and higher survival rates after myocardial infarction (MI) [88]. ANP and CNP levels are elevated in OSTN-Tg mice, thus the cardioprotective effects of osteocrin in this model have been ascribed to the inhibition of NPR-C-mediated natriuretic peptide clearance. Clearly, further investigation is required to understand more about the switch between NPR-C signalling and clearance and if the balance changes in different pathological conditions contributing to cardiac disease (e.g., pressure overload or MI). 

## 9. Coronary Vasodilator Effects of CNP

The first studies of the vascular actions of CNP in the heart were performed in porcine coronary arteries. These early experiments provided the first evidence that CNP exerts coronary vascular relaxation via hyperpolarisation. CNP responses could be inhibited by the potassium channel blockers charybdotoxin and glibenclamide [55]. The same authors also discovered that HS-142-1 inhibits reductions in coronary flow induced by CNP in dogs, suggesting there is a NPR-B/cGMP component of CNP relaxation in the heart [156]. In contrast, studies in rodent Langendorff-perfused hearts showed that CNP and cANF^4-23^ induce reductions in coronary perfusion pressure via the activation of NPR-C and the opening of GIRK channels, in a mechanism analogous to the mesenteric vasculature [157]. Furthermore, increases in CNP peptide could be measured in coronary effluent following stimulation with ACh, suggesting CNP is released as an endothelium-derived hyperpolarising factor (EDHF) by coronary vessels. Intriguingly, in ecCNP KO and NPR-C KO mice, the response to endothelium-dependent vasodilators and flow-mediated dilatation (a shear stress response) are diminished [145]. Thus, CNP may be released during cardiac stress in response to changes in flow. Interestingly, NT-pro CNP levels predict mortality and cardiac readmission in patients with unstable angina, a condition characterised by high wall shear and altered coronary vascular flow [158]. 

## 10. Role of CNP in Ischemia Reperfusion Injury and MI

Microvascular obstruction is a pathological feature of acute MI and frequently occurs despite the restoration of flow to ischaemic tissue following coronary interventions. Two major contributing factors are impaired vasodilation and neutrophil plugging, which lead to mechanical obstruction of the vessels and the release of oxidants and pro-inflammatory mediators [159]. Given the coronary vasodilator capacity of CNP and its release in response to shear stress, it is hypothesised that this vasoactive mediator may improve coronary flow and reduce tissue damage by inhibiting inflammatory cell accumulation and obstruction of the coronary vessels. Data from human studies show that CNP gene expression is elevated in failing ischaemic hearts, suggesting it may play a role in the physiological protective response during MI [160]. The acute effects of CNP during myocardial ischaemia reperfusion (I/R) injury have been studied in isolated hearts, an experimental system devoid of circulating inflammatory cells. In this setting, infusion of CNP attenuates the increase in coronary perfusion pressure during reperfusion, reduces infarct size, and improves left ventricular contractility [157]. The protective effect of CNP in this model is abolished by the NPR-C antagonist M372049 and recapitulated by the infusion of cANF^4-23^. Furthermore, a larger infarct size and poorer functional recovery of the heart has been observed in NPR-C KO animals subjected to the same experimental protocol, intimating that NPR-C activation by CNP is beneficial during I/R [145]. However, it should be noted that the coronary vasodilator responses to CNP are not completely abolished in NPR-C KO mice, suggesting that NPR-B activation may, in part, mediate some of the vasorelaxant effects of CNP in the heart. It is also likely that NPR-B-mediated increases in cGMP/PKG I-signalling contribute to the cardioprotective effects of CNP following ischaemia [161,162].

Patients with pre-existing microvascular dysfunction are more vulnerable to myocardial injury following percutaneous coronary intervention, therefore one might expect that a loss of endothelial-derived CNP would make the heart more susceptible to damage following I/R. However, genetic ablation of CNP from cardiomyocytes results in poorer recovery from I/R injury, whilst deletion of endothelial CNP does not worsen the phenotype [145]. It is possible that cardiomyocyte-derived CNP has a direct effect on contractility following I/R in the isolated heart, however the mechanism involved has not been investigated. It has been postulated that NPR-C coupling to K_ATP_ channels may confer the beneficial effects of CNP during I/R injury as CNP can induce the opening of K_ATP_ which is known to reduce cardiac and metabolic stress during ischaemic injury [162].

The effect of CNP overexpression and knockdown has also been explored in chronic models of MI with the aim of understanding its role in mediating inflammation and cardiac remodelling in the long term. The latest research employing CNP gene silencing in rats demonstrates that abrogation of the endogenous production of CNP by cardiomyocytes results in a larger infarct size following I/R, greater cardiac fibrosis, and an increase in the inflammatory markers TNFα and IL-6 [163]. In contrast, others have reported that cardiomyocyte overexpression of CNP does not affect infarct size but does reduce cardiac hypertrophy and the number of mononuclear infiltrates observed in the myocardium [164]. Similarly, chronic infusion of CNP in a model of permanent coronary artery ligation reduces left ventricular enlargement, collagen deposition, and increases cardiac output [165]. In addition to this, an increase in CNP expression has been reported in the infarct border zone in swine hearts, where it is believed it may contribute to myocardial restoration by increasing capillary density (dovetailing well with the pro-angiogenic actions of the peptide) [166]. Together, these findings suggest that CNP could be a therapeutic target in MI as it is effective at reducing infarct size, cardiac inflammation, and the adverse ventricular remodelling that occurs following MI which may slow the progression of HF. 

## 11. CNP Regulates Heart Rate and Electrical Conduction in the Sinoatrial Node (SAN)

CNP affects heart rate via two mechanisms, the alteration of ionic currents in the SAN, and the modulation of sympathetic drive. It has been reported to induce both positive and negative chronotropic effects in the heart via the modulation of L-type Ca^2+^ currents in the SAN [129,140,167,168]. Under basal conditions or mild stimulation with a β-adrenoceptor agonist, CNP elicits an increase in heart rate and electrical conduction through the SAN. This response is attenuated by the PDE3 inhibitor milrinone, suggesting that the mechanism involves NPR-B/cGMP-mediated inhibition of PDE3 and an increase in cAMP, akin to the positive inotropic effects of CNP observed in myocytes [169]. In contrast, when heart rate is elevated, CNP induces a negative chronotropic effect and decreases conduction velocity within the SAN. NPR-C is believed to mediate this response as cANF^4-23^ reduces the chronotropic effect of isoprenaline but has no effect under basal conditions [170]. The importance of NPR-C signalling in the SAN has been demonstrated in studies using KO mice. Deletion of NPR-C results in SAN dysfunction, prolongation of SAN recovery time, and increased susceptibility to atrial fibrillation [171]. These mice also exhibit atrial fibrosis at baseline which is thought to contribute to aberrant SAN conduction. This is exacerbated in models of heart failure, although treatment with cANF^4-23^ reduces the number of arrhythmias and the changes in electrophysiology [151].

The second mechanism by which CNP modulates heart rate is via the inhibition of cardiacsympathetic neurotransmission in the heart. CNP treatment reduces tachycardia during right stellate (sympathetic) ganglion stimulation in rats and inhibits the release of norepinephrine from isolated atria [172]. Evidence for an endogenous CNP/NPR-B pathway regulating sympathetic activity is demonstrated in transgenic rats with neuron-specific overexpression of a dominant negative form of NPR-B. These animals exhibit elevated heart rates, greater heart rate variability, and frequency domain analyses reveal a higher low-frequency (LF)/high-frequency (HF) ratio, indicative of a shift towards sympathoexcitation [172]. Similar findings have also been reported in mice lacking NPR-C. These animals display a reduction in circadian changes of heart rate, a loss of dynamic changes due to alterations in activity, and a greater LF/HF ratio, suggesting that sympathetic activity is enhanced [173]. Nevertheless, regardless of the receptor that mediates this sympatho-inhibitory effect of CNP, the ability to dampen sympathetic activity in the heart may be an important protective mechanism in diseases characterised by autonomic dysregulation. 

## 12. Current and Future Therapeutics

The past decade has yielded a vast amount of evidence supporting a broad homeostatic role for CNP in maintaining vascular and cardiac function. Moreover, the latest research employing transgenic models has enabled a greater depth of understanding of the key physiological functions of the peptide in the cardiovascular system. Therapeutics designed to bind to the cognate receptors for CNP could have wide-ranging clinical applications in diseases such as hypertension, atherosclerosis, restenosis, critical limb ischaemia, peripheral arterial disease, I/R injury, MI, HF, and heart rhythm disorders. Currently, there are two therapies that target the natriuretic peptide system that have been tested in clinical trials, NEP inhibitors (inhibit the breakdown of natriuretic peptides) and cenderitide (a chimeric NPR-A/NPR-B agonist). NEP inhibitors, used in combination with angiotensin converting enzyme inhibitors (ACEi), have been trialled in patients with hypertension and HF. Initial results were promising, however there was a higher occurrence of angioedema reported in patients on dual treatment compared to ACE inhibitor alone, therefore development was halted [174]. However, the NEP inhibitor sacubitril, given in combination with the angiotensin receptor blocker (ARB) valsartan (LCZ696), has been used with more success. This drug appears to be more efficacious at reducing blood pressure than the currently available ACEi and ARBs, with a similar safety and tolerability profile [175,176]. Furthermore, LCZ696 had impressive results in the PARADIGM-HF trial for the treatment of patients with HF and reduced ejection fraction (EF). The results from the trial showed significantly greater benefits of this combination therapy compared to standard therapy (ACEi treatment alone) [177]. A significant reduction in cardiovascular mortality and heart failure related hospitalization (20%) was reported and the trial was terminated early due to the overwhelming benefit with regard to the primary endpoint. LCZ696 is now licensed as Entresto and is currently being used in a clinical trial (PARAGON-HF) for patients with HFpEF [178]. Given that hypertension is common in this group of patients and the disease is associated with reduced cGMP availability [179], boosting natriuretic levels may be advantageous. Theoretically, NEP inhibition could increase the levels of all natriuretic peptides and enhance their beneficial effects, however, it should be noted that NEP also cleaves other vasoactive peptides, such as bradykinin, so the outcome of this treatment may not be solely down to a reduction of natriuretic peptide degradation. Although, higher levels of cGMP and BNP have been reported in patients receiving LCZ696, indicating that elevated natriuretic peptide levels likely contribute to the protective effect of NEP inhibition. Moreover, CNP is more susceptible than ANP and BNP to NEP degradation, so it may be an important contributor to LCZ696 efficacy [18].

Cenderitide (CD-NP) is a novel ‘designer’ natriuretic peptide that consists of CNP plus the C terminus of *Dendroaspis* natriuretic peptide (isolated from the green mamba snake). It is a dual NPR-A/NPR-B agonist that has been engineered to harness the anti-fibrotic, anti-proliferative, and vascular regenerating properties of CNP and the beneficial renal effects of NPR-A activation [180]. A key benefit of this drug is the fact that it is more resistant to NEP degradation than the native natriuretic peptides [181]. The first clinical target of this drug is HF as it has proven to be efficacious in a rat model of early stage disease in which CD-NP reduces fibrosis and diastolic dysfunction [149]. In addition, CD-NP causes a greater reduction in collagen production by human cardiac fibroblasts than BNP or CNP alone [128,182]. It has been suggested that targeting NPR-B in the heart could potentially be detrimental if NPR-B/cGMP signalling increases adrenergic drive (via PDE3 inhibition) in vivo as it has been shown to do in vitro [138]. Indeed, clinical studies of the PDE3 inhibitor milrinone, demonstrate increased mortality, sudden death, and arrhythmias in HF patients, so the effects of NPR-B agonists on PDE3 activity would need to be investigated thoroughly. However, the first trial in man has shown that Cenderitide is safe, well-tolerated, and causes increases in plasma and urinary cGMP in patients with HF, suggesting that this could prove a promising therapeutic agent in the future [182]. More recently, Burnett et al. have developed other designer natriuretic peptides, such as C53, a long-acting NPR-B activator that is resistant to NEP and has limited interaction with NPR-C which elicits potent anti-fibrotic effects in renal and cardiac fibroblasts [150]. The newest compound in this drug development pipeline, CRRL269, a non-hypotensive activator of NPR-A, is being considered for use in acute kidney injury [183]. 

The rationale for the development of NPR-C agonists came from studies indicating that this receptor mediates a large proportion of the effects of CNP on vascular tone [65,73], in addition, NPR-C mutations are linked to hypertension in GWAS [85]. NPR-C has also been shown to mediate, at least in part, the endogenous effects of CNP in failing hearts, vascular regeneration/angiogenesis, and inflammation. Furthermore, NPR-C is the receptor that is upregulated the most in HF. Targeting NPR-C could also potentially avoid effects on bone development, which are mediated primarily by NPR-B. Thus, small molecule agonists of NPR-C have been designed according to the crystal structure of the receptor bound with CNP [184] and the selective antagonist M372049 [185]. The lead compound 118 has been shown to reduce blood pressure in vivo and relaxes mesenteric arteries in vitro [73]. Furthermore, 118 has high affinity and slow dissociation characteristics at the receptor so it could compete for the clearance function of NPR-C. Accordingly, NPR-C agonists may have the additional benefit of being able to reduce the degradation of all natriuretic peptides and could have broader therapeutic effects than those conferred by NPR-C signalling alone. Further development and optimisation are ongoing.

## 13. Summary

**Figure 1:**
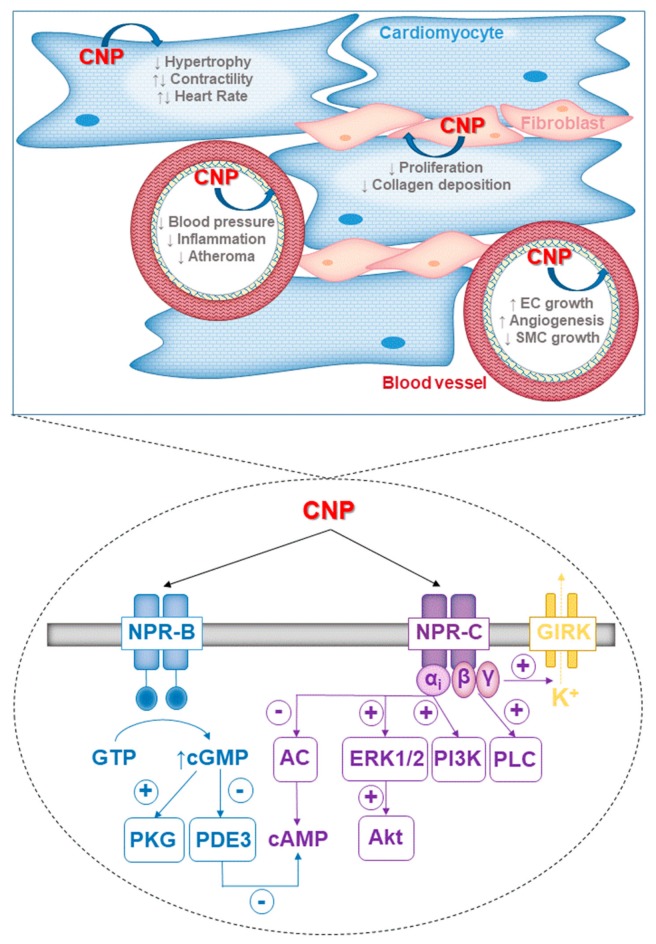
(Patho)physiological functions (upper panel) and signalling pathways (lower panel) activated by CNP in endothelial cells, cardiomyocytes, and fibroblasts. In the heart, CNP reduces cardiac hypertrophy, inhibits fibrosis, and modulates myocyte inotropy and chronotropy. In the vasculature, CNP lowers blood pressure, inhibits inflammation, reduces atherosclerotic plaque deposition, modulates endothelial cell (EC) and smooth muscle cell (SMC) growth, and stimulates angiogenesis. The cellular effects of CNP are mediated via two cognate receptors, NPR-B and NPR-C. NPR-B is a particulate guanylyl cyclase receptor and stimulation results in the production of cGMP and the activation of protein kinase G (PKG) I. NPR-C is G_i_ protein-linked receptor that modulates various intracellular enzymes including adenylyl cyclase (AC), phospholipase C (PLC), extracellular signal-related kinase (ERK) 1/2, phosphoinositide-3-kinase (PI3K), and protein kinase B (Akt). NPR-C activation also triggers the opening of G-protein gated inwardly rectifying potassium (GIRK) channels. Cross-talk occurs between the two receptor signalling pathways via cGMP-mediated inhibition of phosphodiesterase (PDE) 3, the enzyme responsible for the hydrolysis of cAMP in cardiomyocytes.

CNP drives a multitude of cardiac and vascular protective effects via its two cognate receptors, NPR-B and NPR-C. These beneficial actions are mediated by a number of distinct molecular pathways (Figure 1). Pharmacological targeting of NPR-B and/or NPR-C harnesses these salutary functions and holds wide-reaching therapeutic promise for cardiovascular disease.
